# Biology and Pathogenesis of *Staphylococcus* Infection

**DOI:** 10.3390/microorganisms8030383

**Published:** 2020-03-09

**Authors:** Valentina Virginia Ebani

**Affiliations:** Department of Veterinary Sciences, University of Pisa, Viale delle Piagge 2, 56124 Pisa, Italy; valentina.virginia.ebani@unipi.it

Members of the genus *Staphylococcus* still represent a topic of great relevance due to the numerous types of infections they cause in humans and animals.

Staphylococci have emerged as important pathogens for both nosocomial and community-acquired infections in people. For example, USA300 is a predominant community-associated methicillin-resistant *S. aureus* strain; it carries an arginine catabolic mobile element (ACME) which contains potential virulence factors playing a role in bacterial virulence and transmission [1].

Human bloodstream infections are often caused by *S. aureus*. It has been demonstrated that the strains responsible for these types of infection have different virulence genes which influence the bacterial pathogenicity and can be horizontally transferred among bacterial strains [2]. 

*Staphylococcus* members are a severe threat in veterinary medicine, too, causing diseases in farm animals and pets. Bovine mastitis is a costly disease to the dairy industry; intra-mammary infections by *S. aureus* are frequent and strains belonging to ruminant-associated clonal complexes are predominantly involved [3]. 

Staphylococcal infections are documented in fish, too. *Staphylococcus xylosus* infection has been observed in rainbow trout (*Oncorhynchus mykiss*), in which it determines mortality and consequent economic losses in trout fisheries. In particular, *S. xylosus* causes exophthalmia and disrupts the primary immune barrier, which induces secondary bacterial infections in fish under poor conditions [4].

Furthermore, members of this genus have been found in marine mammals living in very cold environment, such as Antarctica. *Staphylococcus delphini* were found in Adélie penguins (*Pygoscelis adeliae*). The report of *S. pseudintermedius* from Weddell seals (*Leptonychotes weddellii*) confirmed its occurrence in all families of the suborder Caniformia [5]. 

In the last years, the incidence of antibiotic-resistant Staphylococcus isolates—both coagulase-positive and coagulase-negative [6]—has increased, becoming a severe problem for human and animal therapies. In this view, in vitro studies have been performed to find new solutions for possible future alternative treatments. Lubowska et al. [7] found that *Kayvirus* bacteriophages are active in vitro against multidrug-resistant (MDR) *S. aureus* isolates. Yang et al. [8] studied a cysteine-capped hydrogel able to absorb and release copper, an ion with the capability of suppressing the growth of staphylococci; in particular, this preparation resulted effective in vitro against *S. aureus* USA300 strain. A study on the lytic activity of cell-wall hydrolases showed that these enzymes have antimicrobial activity in vitro against *Staphylococcus* spp. [9]. Lastly, essential oils, mainly *Origanum vulgare*, *Thymus vulgaris* and *Satureja montana*, showed antimicrobial activity in vitro against *Staphylococcus* spp. strains isolated from dogs with cutaneous infections [10].

Biofilm formation has a huge impact on infection. Some studies added interesting data about this topic. Hiltunen et al. [11] carried out an investigation to better understand the capability of these bacteria to produce biofilm on different clinically relevant materials (borosilicate glass, plexiglass, hydroxyapatite, titanium and polystyrene).

Savjioski et al. [12] described and compared surfaceomes (cell surface proteins) from *S. aureus* cultures grown for prolonged time periods in planktonic and biofilm forms. This study revealed that the phenotypic state of the cells prior to biofilm formation affects the immune-evasion and persistence-related traits of *S. aureus*.

Biofilm-mediated infection is a major cause of bone prosthesis failure, too. In this view, Reigada et al. [13] assess the potential applicability of three previously discovered anti-biofilm compounds to be part of implanted medical devices by testing them on in vitro systems that more closely resemble the clinical scenario.

Additional information related to the pathogenesis of staphylococcal infections are reported in the manuscript by Balraadjsing et al. [14]; the authors confirmed that dendritic cells have an important role in the control and regulation of anti-staphylococcal T cell responses; moreover, they found that dendritic cells internalize *S. aureus* more efficiently than *S. epidermidis*, but do not differ in induction of antigen-specific T cell proliferation.

In conclusion, this special issue has collected studies regarding different aspects of the biology and pathogenesis of *Staphylococcus* infections, with the hope of having aroused the interest of practitioners and researchers operating in human and veterinary medicine.
